# PSMA-Positive Low Malignant Gastrointestinal Stromal Tumor in the Stomach on F-18-PSMA-1007 PET/CT

**DOI:** 10.3390/diagnostics12020227

**Published:** 2022-01-18

**Authors:** Peter Iversen, Allan Kjeldsen Hansen, Thorbjørn Hubeck-Graudal, Lise Medrud, Kirsten Bouchelouche

**Affiliations:** 1 Department of Nuclear Medicine and PET-Centre, Aarhus University Hospital, Palle Juul-Jensens Boulevard 165, 8200 Aarhus, Denmark; kirbou@rm.dk; 2 Department of Nuclear Medicine, Regional Hospital West Jutland, Gammel Landevej 61, 7400 Herning, Denmark; allanhan@rm.dk (A.K.H.); thohub@rm.dk (T.H.-G.); 3 Department of Radiology, Aarhus University Hospital, Palle Juul-Jensens Boulevard 99, 8200 Aarhus, Denmark; lise.medrud@auh.rm.dk

**Keywords:** PSMA, prostate cancer, FDG, polyp, pitfalls, PET/CT, GIST

## Abstract

A 76-year-old man with newly diagnosed high-risk prostate cancer was referred for primary staging with F-18-PSMA-1007 PET/CT. The PET/CT scan showed no lymph node or bone metastases, only localized disease within the prostate gland. Additionally, the F-18-PSMA PET/CT scan showed a PSMA-positive lesion correlating to a polyp located in the body of the stomach on the greater curvature. A prior F-18-FDG PET/CT showed low FDG uptake in the polyp, but this was not reported initially in the written report. The patient had no upper gastrointestinal symptoms. A gastroscopy with biopsies was performed, and the histopathology results showed chronic unspecific inflammation with no granulomas, dysplastic or malignant changes in three out of three biopsies. A repeated gastroscopy with biopsy showed an epithelioid variant of a gastrointestinal stromal tumor (Ki-67 index 2%). A laparoscopic tumor extirpation was planned after radiation treatment in combination with endocrine therapy of the localized prostate cancer. To our knowledge, this is one of very few reported cases of a PSMA-positive gastrointestinal stromal tumor (GIST), and can be added to the list of malignant pitfalls of PSMA PET/CT in prostate cancer patients.

A 76-year-old man with newly diagnosed prostate cancer. The PSA level was 13 μg/L. Gleason 4 + 4 = 8 was found in 11 of 12 biopsies, alongside a cT2c tumor and a prostate volume of 20 ccm. About one month prior to the diagnosis, the patient was referred for an 18-F-FDG-PET/CT for unspecific symptoms, moderate elevation of C-reactive protein level and fever in order to locate infection sites or active inflammation, with the secondary aim of ruling out an underlying cancer. The patient had a medical history of methotrexate-treated seropositive rheumatoid arthritis. The F-18-FDG PET/CT showed localized uptake in the prostate and reactive mediastinal lymph nodes, and low FDG uptake (SUVmax 2.8) in a 35 mm polyp in the body of the stomach (Figure 1, Panel C and D). The latter was not mentioned in either the CT or PET report.

One month later, the patient was referred for primary staging with F-18-PSMA-1007 PET/CT. The scan showed localized disease within the prostate with no sign of involvement of the seminal vesicles, lymph nodes or bones. Additionally, the scan showed a PSMA-positive lesion (SUVmax = 21) in the body of the stomach on the greater curvature, correlating to a 35 mm polyp located close to the pylorus (Figure 1, Panel A and B).

A gastroscopy with biopsies initially showed chronic unspecific inflammation with no granulomas, dysplastic or malignant changes in three out of three biopsies. A repeat biopsy showed an epithelioid variant of a low malignant gastrointestinal stromal tumor (Ki-67 index 2%). A laparoscopic tumor extirpation was planned after radiation treatment in combination with endocrine therapy of the localized prostate cancer.

Increased PSMA expression is seen in most prostate cancers but has also been reported in other malignant and benign conditions [1,2,3]. PSMA PET is now widely used for detecting biochemical recurrence of prostate cancer [4] but is also increasingly used for primary staging of high-risk prostate cancer [5]. To our knowledge, this is one of very few reports [6,7] demonstrating PSMA-positive GIST. The other reported GIST cases were localized in the small bowel and in the gastric fundus. PSMA-PET could potentially be a competitive tracer of F-18-FDG-PET for the staging of GIST. However, a systematic review and meta-analysis by Kim and Lee [8] showed a high pooled FDG sensitivity of 88% in 177 patients across seven studies. In our case, the GIST tumor was not detected in the initial F-18-FDG PET/CT, but only in the following F-18-PSMA PET/CT. GIST tumors can be added to the list of PSMA-positive malignant pitfalls when reporting PSMA PET/CT scans in prostate cancer patients. This is likely due to PSMA binding to endothelial cells of the neovasculature, as seen in other PSMA-positive non-prostate cancers.

## Figures and Tables

**Figure 1:**
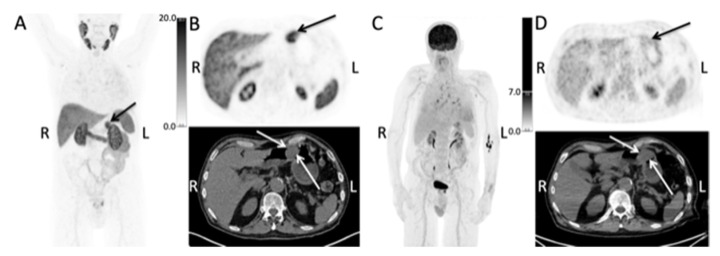
The maximum intensity projection (MIP) of the F-18-PSMA-1007 PET/CT (**A**) show a PSMA-positive lesion located in the upper abdomen (arrow) (R, right side; L, left side). Transaxial images of the of the F-18-PSMA-1007 PET/CT (**B**) show the PSMA-positive polyp in the body of the stomach, as indicated by the arrows. The maximum intensity projection (MIP) (**C**) and transaxial images (**D**) of the prior F-18-FDG PET/CT showed low FDG uptake in the polyp (arrows).

## Data Availability

Not applicable.
